# Identifying patient perceived values during outpatient encounters: an empirical study from Chinese public hospitals

**DOI:** 10.1186/s12913-023-09817-6

**Published:** 2023-08-11

**Authors:** Sha Liu, Yinhuan Hu, Chuntao Lu, Dehe Li, Zemiao Zhang

**Affiliations:** 1 School of Medicine and Health Management, Tongji Medical College, Huazhong University of Science and Technology, No.13 Hangkong Road, Wuhan, 430030 China; 2 Jingmen No. 2 People’s Hospital, Jingmen, China

**Keywords:** Patient perceived value, Outpatients, Visit process, Research model, China

## Abstract

**Background:**

Focusing on patients’ perceived values is essential for patient-centered health care. Only by identifying the patient’s preferred values can we better meet their needs and provide them with valuable medical services. This study aimed to construct and validate a research model to obtain an overall quantification of patient value during outpatient encounters.

**Methods:**

The development of the research model was based on the reviewed literature, and an initial theoretical framework was formed by an expert panel discussion. A scale questionnaire for all the items was adapted from previous research related to patient value, verified using a presurvey, and thus used for data collection for this study. The structural equation model was used to determine and evaluate the research model of the values patients perceived during outpatient encounters.

**Results:**

572 eligible respondents who completed outpatient visits from a typical public hospital in China participated in this study from November 2020 to February 2021. We constructed the patient perceived value (PPV) model to identify core values, which includes eight dimensions and 29 items in terms of functional value (installation, efficiency, price, service quality), emotional value (interactive, control), and social value (accessibility, image) from two subgroups of patient value outside and in the outpatient visit process. Cronbach’s alpha for the whole model was 0.950. The confirmatory factor analysis showed that the PPV model fits well, with a correlation of 0.83 between the two subgroups.

**Conclusion:**

It is essential to recognize the values based on patients’ perceptions and experiences throughout the entire visit process. Our findings offer targeted insights for healthcare administrators, enabling them to holistically optimize outpatient service processes and continually enhance the quality of outpatient medical services from the patient’s perspective.

**Supplementary Information:**

The online version contains supplementary material available at 10.1186/s12913-023-09817-6.

## Introduction

With the homogenization of hospital competition, researchers and practitioners are increasingly focusing on patient value, recognizing that offering patient-oriented medical services is critical for improving hospital service capacity. The visit process and patient experience in outpatient care have a significant impact on patients’ perceived value. By identifying the core value, we can better understand and meet the needs of patients, providing them with valuable medical services. In the context of medical systems, it is crucial to prioritize patient perception when expressing value. Patient perceived value (PPV) refers to the value that patients perceive during their visit, serving as a measure of patient satisfaction and an assessment of healthcare service capabilities from the patient’s perspective. Perceived value is characterized as the result of the customer’s overall evaluation of the benefits and sacrifices made by the received service [1]. Considering patients as customers of the hospital, the theory of customer perceived value can be appropriately applied to medical services. As customers are value-oriented, it is imperative for hospital administrators to define the concept of patient value, which encompasses the perception of the differences between the expected medical value formed during the visit process and the actual medical outcome. [2].

Previous research on patient perceived value has primarily concentrated on the clinical diagnosis and treatment effects of specific diseases collected from particular inpatient specialties [3]. These studies have analyzed patients’ perceived quality and satisfaction with healthcare services [4, 5]. However, few scholars have identified the overall value of the patient experience throughout the entire consultation process. Perceived value measures for outpatients are multidimensional [6–8]. According to Woodruff [9], customers may perceive value differently at the stage of product or service purchase, as well as during or after use. Sweeney [6] developed the Perceived Value Scale (PERVAL), which also considered how customers feel in pre-purchase and post-purchase situations. While several researchers have started discussing the initial concepts of patient value in medical research, there have been few studies focused on constructing research models to identify valuable health services from the patient’s perspective, especially in the context of Chinese public hospital outpatient settings.

Public hospitals serve as gathering locations for medical consultations, with the outpatient department acting as the primary entry point for the majority of patients in China. Outpatient therapy settings are particularly susceptible to doctor-patient disagreements and violence hazards due to the open environment and large patient population [10]. However, in order to explore the professionalization and lean management of hospital outpatient services, it is crucial to identify the core value perceived by patients throughout the entire visit process, rather than focusing on just a portion of it. Accurately defining value enables us to better understand and meet the needs of patients, providing them with valuable medical services.

There is a growing awareness that healthcare services based on patient perception and experience are better suited to meet patient needs. By focusing on the patients’ perceived value and delivering valuable healthcare services, we can optimize the outpatient visit process and enhance the efficiency of patient access. Therefore, we proposed a research framework considering the overall perceived value of patients in outpatient. To achieve this, we conducted an exploratory study to identify the composition and classification of PPV based on previous research and the specific context of Chinese public hospitals. The objective of this study was to construct and validate a research model identifying the value perceived by patients during outpatient encounters, which provided a reference for hospital managers to improve the quality of outpatient medical service.

## A conceptual framework

Zeithaml (1988) [11] proposed the theory of perceived value from the perspective of customers. Perceived value is defined as the overall assessment of the utility of a product or service after weighing benefits and sacrifices. According to Cravens et al. [12, 13], perceived value refers to the ratio or equilibrium between the quality of a product or service and its price. It can be seen as a form of currency used by customers to assess the value of their purchases. However, Bolton and Drew(1991) [14] argued that it is too basic to view value as a trade-off between quality and price. The majority of researchers tend to consider perceived value from a broader perspective, and numerous studies have confirmed that perceived value is a complex concept composed of multiple dimensions [15, 16].

In general, value judgments involve individuals forming opinions or assessments regarding the worth or merit of a specific product or service provider. Hartman(1973) [17] proposed that value involves cognitive and emotional aspects including three aspects: extrinsic value, intrinsic value and system value. Extrinsic value is related to the utilitarian nature of the service event. Intrinsic value represents the emotional appreciation of the service process, which relates to the emotional aspects of the service delivery process. Systemic value relates to the intrinsic relationship among concepts in systemic interaction. Mattsson (1991) [18] adapted the framework developed by Hartman’s formal model to three generic value dimensions, emotional(E), practical(P) and logical(L). Sheth et al. (1991) [19] proposed five dimensions of perceived value based on consumer value theory, that is social, emotional, functional, epistemic and conditional, which illustrated consumer choice behavior. Sweeney and Soutar(2001) [6] described the development of PERVAL which is a scale of measurement of value including six items of quality (functional value), four items of price (functional value), five items of emotional value and four items of social value.

Based on the review of the literature, customer perceived value theory has various dimensional characteristics in different application contexts. The study of PPV, as an extension of customer perceived value, has gradually attracted widespread attention from the medical and academic communities. Based on the theory of customer perceived value, O’Connor and Shewchuk [20] constructed a patient perceived value model in the medical environment from four dimensions, that is medical reliability, medical responsiveness, medical indemnification and medical empathy. In healthcare, Cengiz and Kirkbir [21] believed that PPV indicators consist of various dimensions, such as functional value (installation, service quality, price, professionalism), emotional value (novelty, control), social value and others. Pan and Chen [22] proposed that the indicators of PPV can be divided into five dimensions: quality, emotion, price, reputation and accessibility, with medical quality being the most important dimension, the emotion dimension ranking second, and facilities and price being paid much less attention. Sower et al. designed the Key Quality Characteristics Assessment Scale for Hospitals (KQCAH) based on the SREVQUAL scale, which consists of eight dimensions reflecting patients’ perceived value (respect and care by hospital staff, effectiveness and continuity of services, suitability of facilities, information, cost, food service, first impressions, and staff diversity) [23]. In the mHealth environment, several studies have noted the diverse nature of patient perceived value, with the doctor-patient interaction emerging as a crucial source of value. Influential factors on patients’ perceived value include emotional needs, time costs, pain relief, and doctor-patient trust [24, 25]. Previous studies have examined the association between perceived value and patient loyalty and satisfaction [26–28]. While some studies have identified service quality, emotional value, and professionalism as key drivers of perceived value, there is still a gap in exploring patient perceived value within the broader context of the entire patient experience.

**Fig. 1:**
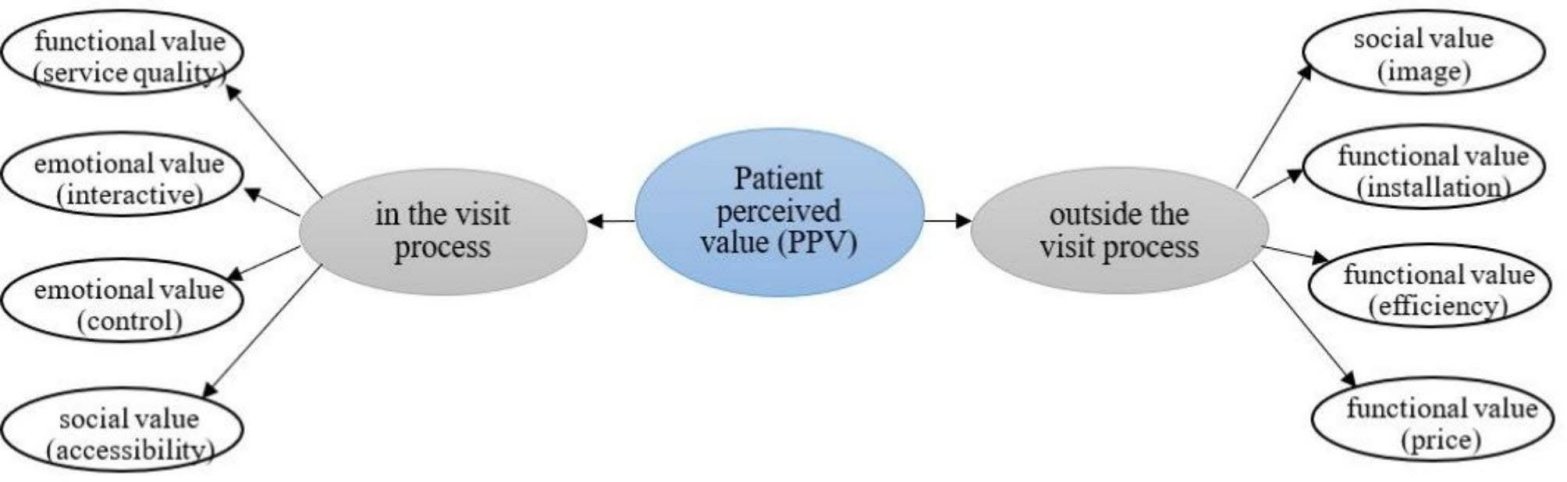
The conceptual framework of patient perceived value

Through the above study, we found that the researchers stated three main dimensions of perceived value: functional value, emotional value and social value [1]. In the healthcare sector, the functional dimension typically refers to the rational and economic evaluation of people, whereas the emotional and social dimensions mainly reflect the subjective feelings generated by the diagnosis and treatment experiences. In our study, functional values refer to the tangible and measurable aspects of the services received by patients. This includes their evaluation of the hospital environment, facilities, waiting time, price of services outside the outpatient visit process, and the quality of services during the outpatient visit process. Emotional values are related to the patient’s emotional responses and experiences during their interactions with healthcare professionals, including the doctor-patient interaction and risk control during the visit. Social values refer to the patient’s perception of the hospital’s reputation, image, and accessibility of healthcare services. Therefore, we summarized and proposed the PPV model as a multidimensional structure comprising eight dimensions: social value (image), functional value (installation), functional value (efficiency), functional value (price), functional value (service quality), emotional value (interactive), emotional value (control), social value (accessibility). Additionally, through expert panel discussions, we further divided PPV into two subgroups: outpatient value outside the visit process and outpatient value in the visit process. This initial theoretical framework is depicted in Fig. 1.

## Methods

### Survey instrument design

The questionnaire scale items for all eight PPV dimensions were adapted from a thorough review of the literature to develop. We referenced previous studies on customer perceived value dimensions to guide the design of the scale items. Functional value, which represents the quality and level of hospital outpatient medical services provided, can be further divided into four components: installation, efficiency, price, and service quality. The scale items for functional value (installation and price) were adapted from the GLOVAL (global purchase perceived value) scale provided by Sa´nchez et al. [29]. Functional value (efficiency and service quality) were measured using the scale developed by Mathwick et al. [30] and Gallarza, Saura [31], supplemented by related research from Chinese scholar Jie Zhao [32]. Emotional value dimensions including interactive and control were measured via scales developed by Otto and Ritchie [33], whereas social value items were adapted from Fengchuan Pan [22], Haixiao Chen [34] and Ralston [35] (see Appendix).

To improve the validity of the initial item selection, we organized a focus group discussion involving experts, academics and physicians to confirm the items and evaluate the face validity and content validity of the items. Based on the feedback received, we removed redundant items and revised the wording of incomprehensible items to make the questionnaire understandable in important ways. We conducted a pilot study with the modified questionnaire by a random sample of 150 patients to further determine the item list. The data from this pre-test were analyzed, and items that showed a correlation value of less than 0.3, indicating poor association with PPV items, were selected for deletion. The final version of the questionnaire with the effort contained 8 dimensions and 34 items for evaluation.

### Data collection

The survey was conducted on a sample of 600 outpatients from the outpatient department of a tertiary general public hospital including three branches in Wuhan city, Hubei province, which was the typical institution that received patients. The sample size determined by general guidelines for 1:10 of item-to-response ratios was deemed sufficient for a robust analysis of the proposed model [36]. Respondents selected by convenience sampling were asked to consider their perceptions of public hospital services both outside and in the outpatient visit process. Participants meeting the inclusion criteria were those who had completed the outpatient visit procedure and agreed to join the study voluntarily. All the subjects were required to be at least 18 years old and possess sufficient Chinese skills to answer a written questionnaire. The items were measured with a 5-point Likert scale. Respondents were asked to estimate how much they agree with each item on the scale (strongly disagree:1, disagree:2, neutral :3, agree:4, strongly agree:5). Overall, the data collection period is from November 2020 to February 2021.

In our study, we identified 28 samples that were deemed inappropriate for further analysis and therefore excluded. The exclusion criteria employed consisted of incomplete questionnaire responses, lost questionnaires due to survey process interruptions, omitted information or options, and nonsensical or inconsistent responses. Specifically, we excluded questionnaires that failed to provide answers to key questions or contained obvious logical errors. After applying these criteria, a total of 572 valid questionnaires were obtained and coded for subsequent data analysis. Furthermore, the reliability of the patient perceived value construct was assessed using Cronbach’s alpha coefficient, resulting in a high value of 0.963, indicating that the measurement scale was reliable. The applicability of factor analysis is determined by the Kaiser-Meyer-Olkin (KMO = 0.962) measure of sampling adequacy and Bartlett’s test of sphericity (P < 0.001).

### Data analysis

SPSS21.0 was employed to perform descriptive analysis, correlation analysis, and exploratory factor analysis on the collected data, and the construct validity of the questionnaire was conducted with AMOS17.0. The reliability and validity of the questionnaire results were tested. If Cronbach’s alpha coefficient is above 0.7, the reliability of the scale is considered acceptable [37]. Kaiser–Meyer–Olkin(KMO) test and Bartlett sphericity test were performed to determine whether factor analysis was appropriate. In this study, half of the 286 questionnaires were randomly selected for exploratory factor analysis(EFA), and the other half were used for confirmatory factor analysis(CFA).

EFA was conducted by using principal component analysis to extract common factors and performing oblique rotation (promax) to increase the interpretability of factors and determine the underlying dimensions of the patient’s perceived value construct. For further study, we eliminated items with low factor loadings (≤ 0.4) and extensive cross-loadings on other factors. We performed CFA based on the final EFA results to verify the hypothesized relationship between latent variables and items.

The construct validity of the model was tested using the goodness-of-fit indicators, such as *χ*^2^/*df*, root mean square error of approximation(RMSEA), Normed Fit Index(NFI), Tucker-Lewis Index(TLI), and goodness-of-fit index(GFI), along with the standardized root mean square residual(SRMR). In general, if χ^2^/df < 3, RMSEA < 0.08, TLI > 0.90, NFI > 0.90, GFI > 0.90, and SRMR < 0.05, it indicates that the goodness-of-fit index is reasonable and acceptable [38, 39].

We assessed the reliability of our scale by calculating its composite reliability(CR), considering a CR value greater than 0.70 as indicative of strong consistency among latent construct indicators [40]. To assess convergent validity, the average variance extracted(AVE) was used as an indicator of the average level of precision for each item within the scale. The AVE of each construct and all the standard loadings should be greater than 0.50. [41] Discriminant validity is evident if the AVE are greater than the squared correlation values between that construct and any other constructs [42]. The internal consistency of the scale was estimated by Cronbach’s alpha coefficient with a good value higher than 0.7. [43].

## Results

### Descriptive results

A total of 572 eligible respondents were included in the analysis. The results of the descriptive analysis of demographic characteristics were shown in Table 1. Among the participants in this study, 60.1% were female, aged from 18 to 81 years (mean ± SD, 32.6 ± 11.1). The overall level of education was relatively high, with 69.5% of respondents having received college education or above.

**Table 1:** Characteristics of respondents (N = 572)

Characteristics	Frequency (n)	Percentage (%)
Sex		
Male	228	39.9
Female	344	60.1
Age		
18–20	31	5.4
21–40	440	76.9
41–60	86	15.0
≥ 61	15	2.6
Residence		
Rural	350	61.2
Urban	222	38.8
Educational level		
Master’s degree or above	58	10.1
Bachelor /college degree	340	59.4
Technical secondary school/senior high school	119	20.8
Junior middle school or below	55	9.6
Outpatient Type		
General Clinic	122	21.3
Specialist Clinic	450	78.7
Medical speciality		
Surgery	94	16.4
Internal Medicine	212	37.1
Gynaecology	89	15.6
Others	177	30.9

### Patient perceived value outside the visit process

The dimension of patient perceived value outside the visit process incorporated the patient’s experience before and after the consultation of public hospitals outpatient. A total of 20 variables were included in the questionnaire construction of the out-of-process dimension. Some of the items (A11, A12, A13) of the patient’s perceived value construct were removed, because of low loading (< 0.40) or cross-loadings on other common factors that did not clearly reflect a latent variable. As a result, four factors were derived from the PPV outside the visit process items (eigenvalue > 1), explaining 63.7% of the variance (see Table 2). The results of EFA were factor 1: social value (image), factor 2: functional value (installation), factor 3: functional value (efficiency) and factor 4: functional value (price).

The measurement model for dimensions of PPV outside the visit process based on the EFA screening results was used for model validation by CFA. The modification index showed that there was a cross-load between A7 and A8, so item A8 was removed. The measurement model fit indices constructed by CFA met the requirements, with χ^2^/df = 2.974(< 3), RMSEA = 0.059(< 0.08), GFI = 0.942(> 0.90), NFI = 0.932(> 0.90), TLI = 0.939(> 0.90), SRMR = 0.046(< 0.05). Besides, convergent validity was verified for the above four dimensions with 16 items, and the AVE values of all items were greater than 0.5 and CR values greater than 0.7. Therefore, the model has good convergent validity and combined reliability. Values of 0.758 or higher for Cronbach alpha indicated good internal consistency (see Table 3). In the subgroup analysis of the factors related to the outside visit process, it was observed that the correlation coefficients of the factors ranged from 0.512 to 0.722, which were all smaller than the square root of the AVE values of the corresponding factors. The results indicated that the discriminant validity among the latent variables in the model was good.

### Patient perceived value in the visit process

The questionnaire on patient’s perceived value in the visit process included 14 items, and item B10 was removed because of cross-loading. Four common factors were finally extracted through EFA with eigenvalues exceeding 1, namely factor 5: functional value (service quality), factor 6: emotional (interactive), factor 7: emotional value (control), and factor 8: social value (accessibility), with a cumulative explained variance of 77.9% (Table 2).

Table 3 showed the CFA findings of the measurement model for dimensions in the patient visit process. All the goodness-of-fit indicators met the requirements, with χ^2^/df = 2.796(< 3), RMSEA = 0.056(< 0.08),GFI = 0.961(> 0.90),NFI = 0.973(> 0.90),TLI = 0.974(> 0.90),SRMR = 0.025(< 0.05). All the CR and AVE values were higher than required, and the Cronbach’s alpha coefficients for all the factors were 0.766 or higher indicating good internal consistency. The subgroup in the visit process showed that the correlation coefficients among the factors ranged from 0.663 to 0.780, all of which were smaller than the square root of the AVE values of the corresponding factors, indicating acceptable discriminant validity.

**Table 2:** EFA results of patient perceived value with item loadings of > 0.4

Items	Factor 1	Factor 2	Factor 3	Factor 4	Factor 5	Factor 6	Factor 7	Factor 8
*Dimensions outside the visit process*								
A1	0.790							
A2	0.750							
A6	0.619							
A7	0.572							
A3		0.758						
A4		0.794						
A5		0.774						
A9		0.511						
A10.1			0.647					
A10.2			0.769					
A10.3			0.776					
A10.4			0.655					
A10.5			0.578					
A8			0.457					
A14				0.759				
A15				0.837				
A16				0.707				
*Dimensions in the visit process*								
B1					0.655			
B2					0.664			
B3					0.793			
B4					0.755			
B5						0.605		
B6						0.789		
B7						0.718		
B8							0.649	
B9							0.776	
B11								0.569
B12								0.813
B13								0.801
B14								0.715

**Notes**. Factor1: social value (image), Factor2: functional value (installation), Factor3: functional value (efficiency), Factor4: functional value (price), Factor5: functional value (service quality), Factor6: emotional value (interactive), Factor7: emotional value (control), Factor8: social value (accessibility)

**Table 3:** CFA results of the measurement model of patient perceived value

Items and constructs	Standard loading	AVE	CR		Cronbach’s alpha
***Patient perceived value outside the visit process***					
Factor 1		0.519	0.812		0.758
A1. hospital reputation and popularity	0.711				
A2. doctor authority	0.738				
A6. advanced equipment	0.684				
A7. informative access procedures	0.746				
Factor 2		0.585	0.850		0.838
A3. environmental cleanliness	0.726				
A4. comfort and quietness	0.816				
A5. reasonable space layout	0.769				
A9. medical guide signs	0.745				
Factor 3		0.522	0.850		0.827
A10.1 short registration time	0.667				
A10.2 short payment time	0.773				
A10.3 short drug getting time	0.719				
A10.4 short time to obtain medical reports	0.718				
A10.5 Short waiting time	0.732				
Factor 4		0.554	0.785		0.776
A14. reasonable charges	0.876				
A15. affordable medical costs	0.631				
A16. good service for price	0.705				
Goodness-of-fit indicator					
χ^2^/df	2.974				
RMSEA	0.059				
GFI	0.942				
NFI	0.932				
TLI	0.939				
SRMR	0.046				
***Patient perceived value in the visit process***					
Factor 5		0.702	0.904	0.907	
B1. physician efforts to understand needs	0.837				
B2. Professional treatment	0.819				
B3. Courteous, polite and respectful	0.833				
B4. Serious, responsible and trustworthy	0.863				
Factor 6		0.582	0.807	0.804	
B5. understandable medical advice	0.789				
B6. enough time for physician-patient communication	0.765				
B7. participate in treatment programs	0.733				
Factor 7		0.614	0.761	0.766	
B8. inform risk and seek consent	0.792				
B9. patient privacy	0.775				
Factor 8		0.653	0.883	0.890	
B11. safe and reliable medical services	0.814				
B12. prevention and health promotion	0.785				
B13. promote healthy lifestyle	0.787				
B14. received the desired service	0.844				
Goodness-of-fit indicator					
χ^2^/df	2.769				
RMSEA	0.056				
GFI	0.961				
NFI	0.973				
TLI	0.974				
SRMR	0.025				

AVE: Average Variance Extracted

CR: Composite Reliability

### Patient perceived value modelling

**Fig. 2:**
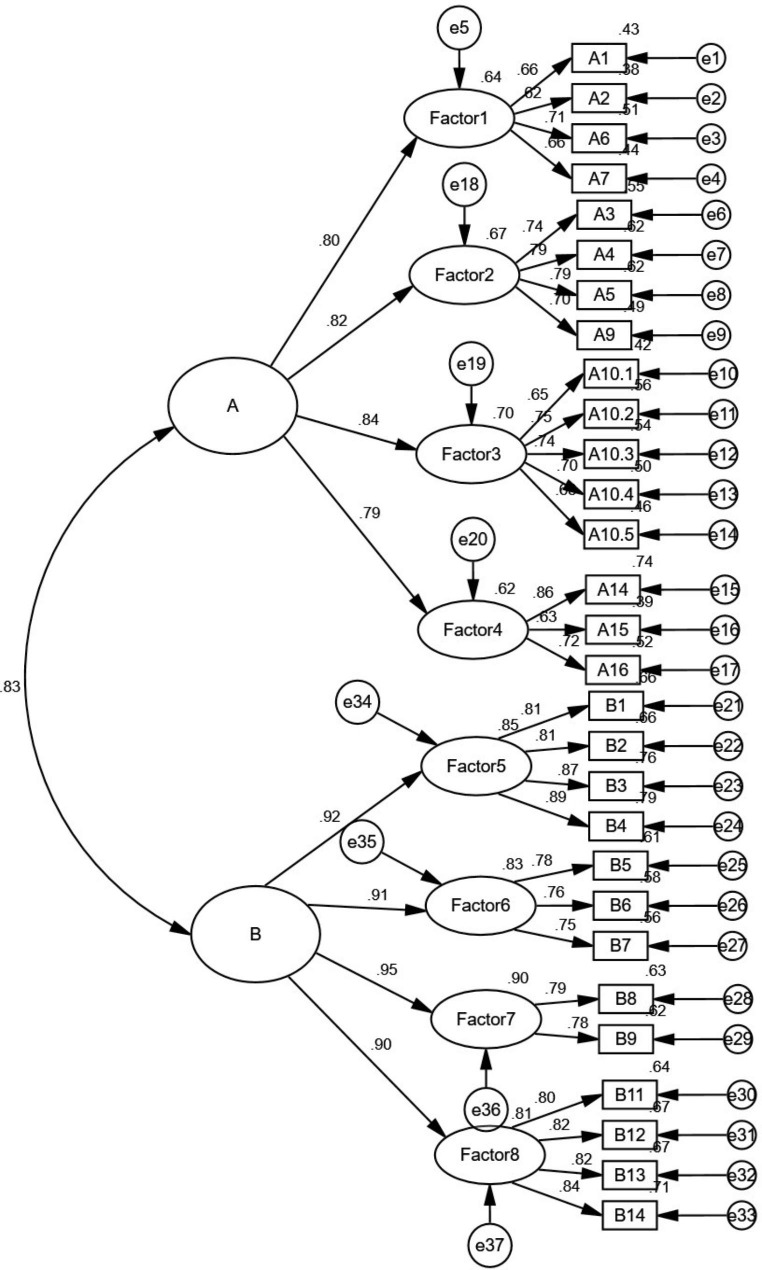
Structural equation model for patient perceived value (subgroup A: patient perceived value outside the visit process; subgroup B: patient perceived value in the visit process)

The final structural equation model (SEM), comprising 29 items, was used to examine PPV using the entire sample. This encompassed subgroup A, representing the dimensions of PPV outside the visit process, as well as subgroup B, representing the dimensions of PPV within the visit process. The Cronbach alpha coefficient for the entire PPV scale reached 0.950, indicating that the scale has good internal consistency reliability, and can effectively measure the concept of PPV. All standardized parameter estimates were significant (P < 0.01), indicating that the sample data support the proposed conceptual model presented in Fig. 2. The Chi-square value of the model was 892.656, P < 0.01, *χ*^*2*^/df = 2.974(< 3), RMSEA = 0.059(< 0.08), GFI = 0.942(> 0.90), NFI = 0.932(> 0.90), TLI = 0.939(> 0.90), SRMR = 0.046(< 0.05). The fit indices for the proposed model were all acceptable showing good fitness.

The items in the PPV model had high factor loadings, with a correlation of 0.83 between subgroup A and subgroup B. Subgroup B had a higher path coefficient than subgroup A, indicating its stronger impact on the overall construct. In the dimensions of PPV outside the visit process, factor 3 functional value (efficiency) had the highest path coefficient of 0.84 among functional values and social values. In the dimensions of PPV in the visit process, factor 7 emotional value (control) had the highest path coefficient of 0.95 among functional values, emotional value and social value. Hence, the research model comprised functional value, emotional value and social value during the patient visiting flow in outpatient was acceptable.

## Discussion

In our study, we developed a PPV model for outpatients in Chinese public hospitals, which enlarged the existing knowledge of patient value. The research model comprehensively assessed core values patients preferred during outpatient encounters, which consisted of eight distinct dimensions in terms of functional value (installation, efficiency, price, service quality), emotional value (interactive, control), and social value (accessibility, image). Unlike previous constructs, our construction divided PPV into two subgroups, A and B, representing patients’ perceived value outside and in the visit process, respectively. Items of subgroup A captured the patient’s evaluation of the experience following a doctor’s visit, as well as their intention to revisit the hospital’s outpatient service. Items of subgroup B focused on the patient’s perception during the outpatient visit itself.

The results revealed that the dimensions of PPV outside the visit process included functional value and social value, with functional value (efficiency) being the primary factor influencing PPV. This can be attributed to the overcrowding of public hospitals in China, particularly in the outpatient departments of comprehensive tertiary hospitals. Patients often spend a significant amount of time waiting in line, with limited opportunities to communicate with doctors about their health conditions due to the complex visit flow. According to several empirical investigations, patient waiting time for treatment and medical convenience are related to PPV, so adequate time extent and perceived convenience should be taken into consideration [44, 45]. Another major factor in determining patient perceptions was social value (image), such as hospital reputation and physician authority, which were known and perceived by patients before they received health care [46]. Furthermore, the environment of the hospitals and the price of medical services could affect patient perceptions. Access to clean and well-maintained facilities, modern equipment and technology, and qualified medical staff can all contribute to a positive patient experience. Conversely, a crowded or dirty hospital environment, outdated equipment, or unprofessional staff can lead to negative perceptions [45]. Patients will weigh the cost of healthcare services against their perceived benefit to make informed and cost-effective decisions [8]. Ultimately, the key to delivering value to patients lies in providing high-quality services that meet their needs at a reasonable price, while also demonstrating a commitment to patient satisfaction and well-being [47].

During the process of visiting a doctor, the patient’s experience primarily stems from their interaction with the healthcare providers. Therefore, compared to the aspects outside the outpatient visit process, the dimensions of emotional value (control and interactive) are added to the PPV within the visit process, which may have a greater impact on patients’ perceptions [21]. Patient safety is a primary concern for both patients and doctors during clinic visits. Physicians play a crucial role in controlling behaviours, such as providing information about risks and ensuring patient privacy, which helps to alleviate the negative impact of information asymmetry between physicians and patients. Positive communication and interaction between physicians and patients were conducive to promoting a harmonious physician-patient relationship. Hospitals can achieve reliable patient satisfaction and earn patient loyalty by consistently providing a high level of value to their customers [48]. Generally, the quality of medical services is one of the factors strongly perceived by patients, and physicians should be polite and responsible to make patients feel that they have received a good medical experience, which is consistent with relevant research findings [20, 26, 27, 49]. The attitude and competence level of doctors not only affect patients’ trust and satisfaction with the doctor but also directly affect the patient’s impression and reputation of the hospital [32]. Therefore, it is crucial for doctors to focus on improving their professional knowledge and skills, actively communicating and problem-solving, and providing patients with a reassuring and comfortable medical experience.

The construction and validation of the PPV model in our study made a significant contribution to the theory and practice. It is widely acknowledged that patient satisfaction is determined by the medical experience and feelings [50]. The construct of patient value, which influences favorable hospital behaviours such as positive image, service quality, and even price sensitivity, could be highlighted [21, 34]. Patient-centered care is an approach that emphasizes meeting the individual needs and preferences of patients, and this study shows that patients prioritize different values when seeking healthcare services. By identifying the functional, emotional, and social values that patients consider important, healthcare providers can tailor their services to better meet patient needs. Hospital managers seeking to enhance patient satisfaction and loyalty should prioritize these core values that patients consider when evaluating their experience [49]. By placing a greater emphasis on patient value, hospitals can ensure that patients perceive their visits as valuable and beneficial, resulting in improved patient outcomes and a stronger reputation in the healthcare industry.

Meanwhile, our study can serve as a guide for policymakers in optimizing outpatient services and creating a more streamlined and efficient workflow, which ultimately leads to better medical service quality overall. The findings provided a framework incorporating patient perceptions and experiences for assessing healthcare quality, which may be extended to hospitals and healthcare systems in other countries to assess their quality of care and identify areas for improvement. Based on the PPV model, healthcare organizations can improve patient outcomes and satisfaction by prioritizing areas that need attention and allocating resources to address them.

### Limitations

There are several limitations to this study. Firstly, the survey sample was limited to a single hospital, which was chosen as a representative public hospital in China. To obtain a more comprehensive understanding of patient value, it is important to validate the results across different regions and various types of public hospitals. Secondly, there is a possibility of self-selection errors in patients’ perceived value due to the different educational levels of the population. Differences in patient background characteristics may result in different perceived outcomes; thus, researchers should be aware that the PPV model should be adapted locally to make it more universal and understandable by groups with lower levels of education [28]. Furthermore, further research should explore the relationship between patient values and physicians’ behaviour, as well as analyze the underlying causes of core values using qualitative methods to extend our conclusions.

## Conclusions

This study developed the PPV model for outpatients in Chinese public hospitals and demonstrated the latent construct for the value patient perceived throughout the entire visit process. The research model, which incorporates patient value both outside and within the visit process, demonstrates good reliability and validity, enabling a comprehensive assessment of patient value. Hospital administrators should prioritize the core values that patients care about and strive to deliver valuable services that meet their needs. The findings offer targeted guidance on holistically optimizing the outpatient service process and continuously improving the quality of outpatient medical services, thereby building a patient-oriented healthcare service system to promote public health.

### Electronic supplementary material

Below is the link to the electronic supplementary material.

Supplementary Material 1: Appendix. The initial PPV scale and literature sources in the questionnaire construction.

## Data Availability

The data that support the findings of this study are available on request from the corresponding author. The data are not publicly available due to privacy or ethical restrictions.
