# Supplementary material for: Identifying patient perceived values during outpatient encounters: an empirical study from Chinese public hospitals
Source: BMC Health Serv Res. 2023 Aug 11;23:852. doi: 10.1186/s12913-023-09817-6 (PMC10422723; doi:10.1186/s12913-023-09817-6)
Supplement: Supplementary file 1 — Supplementary Material 1: Appendix. The initial PPV scale and literature sources in the questionnaire construction. [file 12913_2023_9817_MOESM1_ESM.docx]

**Appendix.** The initial PPV scale and literature sources in the questionnaire construction

| **Construct** | **Items** | **Sources** |
| --- | --- | --- |
| Social Value (image) |  | Haixiao Chen and Hui Qian (2010); Ralston (2003) |
| A1 | Hospital reputation and popularity |  |
| A2 | Doctor authority |  |
| A6 | Advanced equipment |  |
| A7 | Informative access procedures |  |
| Functional Value (installation) |  | Sa´nchez et al (2006) |
| A3 | Environmental cleanliness |  |
| A4 | Comfort and quietness |  |
| A5 | Reasonable space layout |  |
| A9 | Medical guide signs |  |
| Functional Value (efficiency) |  | Mathwick et al. (2002);  Jie Zhao (2020) |
| A10.1 | Short registration time |  |
| A10.2 | Short payment time |  |
| A10.3 | Short drug getting time |  |
| A10.4 | Short time to obtain medical reports |  |
| A10.5 | Short waiting time |  |
| A8 | Convenient procedure |  |
| Functional Value (price) |  | Sa´nchez et al (2006);  Haixiao Chen(2010) |
| A14 | Reasonable charges |  |
| A15 | Affordable medical costs |  |
| A16 | Good service for price |  |
| Functional Value (service quality) | | Gallarza Saura (2006) |
| A11 | Clean and tidy medical staff |  |
| A12 | Provide service in a timely manner |  |
| A13 | Approachable and easy to contact |  |
| B1 | Physician efforts to understand needs |  |
| B2 | Professional treatment |  |
| B3 | Courteous, polite and respectful |  |
| B4 | Serious, responsible and trustworthy |  |
| Emotional Value (interactive) |  | Otto and Ritchie (1996) |
| B5 | Understandable medical advice |  |
| B6 | Enough time for physician-patient communication |  |
| B7 | Participate in treatment programs |  |
| Emotional Value (control) |  | Otto and Ritchie (1996) |
| B8 | Inform risk and seek consent |  |
| B9 | Patient privacy |  |
| B10 | Harmonious doctor-patient relationship |  |
| Social Value(accessibility) |  | Fengchuan Pan and Chishan Chen (2004), |
| B11 | Safe and reliable medical services |  |
| B12 | Prevention and health promotion |  |
| B13 | Promote healthy lifestyle |  |
| B14 | Received the desired service |  |
